# Association of *PON1* Haplotypes (Q192R and L55M) with the Severity of Coronary Atherosclerosis

**DOI:** 10.3390/ijms27167241

**Published:** 2026-08-14

**Authors:** Mariola Rychlik-Sych, Małgorzata Barańska, Michał Dudarewicz, Jacek Owczarek

**Affiliations:** Department of Hospital Pharmacy, Laboratory of Pharmacogenetics, Chair of Biopharmacy, Medical University of Lodz, 90-151 Łódź, Poland; malgorzata.baranska@umed.lodz.pl (M.B.); michal.dudarewicz@umed.lodz.pl (M.D.); jacek.owczarek@umed.lodz.pl (J.O.)

**Keywords:** atherosclerosis, haplotype, paraoxonase 1, polymorphisms

## Abstract

Enzyme paraoxonase 1 (*PON1*) plays a protective role against atherosclerosis by preventing the oxidation of low-density lipoproteins (LDL). Polymorphisms in the *PON1*, particularly Q192R and L55M, have been shown to affect the enzyme activity and, consequently, the cardiovascular risk. The present study aimed to evaluate the association between *PON1* haplotypes comprising the Q192R and L55M polymorphisms and the severity of coronary atherosclerosis in a Polish population. The retrospective study involved 282 individuals of both sexes, divided into two groups following coronarography. The study group included patients after PCI (percutaneous coronary intervention) with stent implantation or patients who qualified for CABG (coronary artery bypass graft), with stenoses of minimum 70% (*n* = 140), whereas the control group included those after coronarography and without essential lesions in coronary vessels (*n* = 142). The Q192R *PON1* polymorphism was identified by PCR-RFLP. The haplotypes 192R-55L and 192R-55M have been shown to be statistically significantly associated with a higher risk of atherosclerosis that requires PCI/CABG (OR = 2.06, 95% CI: 1.29–3.28, *p* = 0.0022; OR = 1.77, 95% CI: 1.17–2.69, *p* = 0.007; respectively). Furthermore, the risk of atherosclerosis requiring PCI or CABG was 53% lower in patients with the 192Q-55L haplotype (OR = 0.47, 95% CI: 0.34–0.66, *p* = 0.000013). Haplotypes 192R-55L and 192R-55M may increase the risk of atherosclerosis requiring PCI/CABG, whereas the 192Q-55L haplotype may lower the risk.

## 1. Introduction

Atherosclerosis, the major cause of cardiovascular diseases, is characterized by progressive plaque formation resulting in angiostenosis and restrictions in the blood flow. One of the key elements in the pathogenesis of atherosclerosis is the oxidation of low-density lipoproteins (LDLs), which leads to their uptake by macrophages and formation of foam cells. Oxidized LDLs (ox-LDLs) more readily penetrate the vascular wall, promote inflammation, induce further endothelial damage and contribute to atheroma development [1,2]. Paraoxonase 1 (*PON1*) is an enzyme that plays a major role in preventing the oxidation of LDL to its more atherogenic form, ox-LDL. *PON1* catalyzes the hydrolysis of phospholipid peroxides, hydroxides of cholesterol esters, hydrogen peroxide and oxidized fatty acid residues in lipoproteins, macrophages and plaques [3]. It also hydrolyzes homocysteine thiolactone, a homocysteine derivative that is a proven risk factor for cardiovascular disease. The thiolactone can damage proteins through homocysteinylation of lysine residues, thereby promoting LDL accumulation and macrophage activation [1]. Other forms of *PON1* activity involve hydrolysis of phosphorus organic compounds (parathion, diazinon, chlorpyriphos), as well as nerve agents, including soman and sarin, and platelet-activating factor (PAF). *PON1* substrates also include lactones such as 3-hydroxy-3-methylglutaryl-coenzyme A (HMG-CoA) reductase inhibitors [4].

So far, the activity and concentration of *PON1* have been shown to be affected by genetic polymorphisms, which may have an impact on susceptibility to atherosclerosis [5]. The *PON1* gene is found in the 7q21.3-q22.1 region. Its expression is regulated by various agents, including genetic polymorphisms, environmental factors and drugs. Two polymorphisms, i.e., Gln192Arg (Q192R, rs662) and Leu55Met (L55M, rs854560), are responsible for changes of amino acids in positions 192 and 55. In the former case, the presence of glutamine in position 192 determines the Q phenotype and the presence of arginine determines the R phenotype. These two alloenzymes differ in the affinity and activity to substrates. Some of them, e.g., paraoxon, are hydrolyzed more rapidly by the R alloenzyme, whereas others, such as phenyl acetate, sarin and soman, are hydrolyzed more rapidly by the Q alloenzyme. The 192Q allele appears to confer protection against oxidation in the context of atherosclerosis [6,7]. In the case of the L55M polymorphism, the presence of leucine is associated with increased activity of the enzyme. The highest activity of paraoxonase 1 is found in individuals with phenotypes 192RR and 55LL, whereas patients with phenotypes 192QQ and 55MM exhibit the lowest activity. Interestingly, the strongest protective potential of *PON1* preventing the oxidation of LDL has been observed in individuals with phenotypes 192QQ and 55MM [8,9].

There are studies that demonstrate associations of the Q192R and L55M *PON1* polymorphisms with the risk of cardiovascular diseases, including atherosclerosis, coronary artery disease, stroke and heart failure. However, the findings remain inconsistent and appear to vary across populations. Identification of haplotypes may help elucidate the observed discrepancies. Therefore, the aim of the present study was to assess the influence of haplotypes for two single nucleotide polymorphisms, Q192R (rs662) and L55M (rs854560), in the gene *PON1* encoding paraoxonase 1 on the severity of atherosclerosis in the Polish population.

## 2. Results

The study group (group A) included 140 patients (57 women and 83 men) who had undergone percutaneous coronary intervention (PCI) or had been referred for coronary artery bypass graft (CABG) due to coronary artery stenosis of at least 70%. The control group (group B) included 142 individuals (78 women and 64 men) after coronary angiography without significant stenoses in coronary vessels. The median age of patients in the study group (65.7 yrs.; min-max: 45.0–88.0 yrs.) was similar to that in the control group (64.3 yrs.; min-max: 40.0–89.0 yrs.; Table 1).

There were no statistically significant differences in terms of medians of individual lipid profile parameters between the study group and the control group: total cholesterol—162.5 mg/dL vs. 171.0 mg/dL, LDL-C—80.0 mg/dL vs. 84.0 mg/dL, triglycerides (TG)—132.0 mg/dL vs. 110.0 mg/dL, HDL-C—53.0 mg/dL vs. 58.0 mg/dL, and non-HDL-C—107 mg/dL vs. 112.2 mg/dL (Table 1).

The frequencies of *PON1* genotypes and alleles for the Q192R and L55M polymorphism are given in Table 2. It was shown that the relative risk of atherosclerosis requiring PCI/CABG, expressed as the odds ratio (OR), in individuals with the 192RR genotype was 2.50 (95% CI: 1.40–4.46, *p* = 0.002). The 192R allele was more frequent in group A than in group B (45.7% vs. 27.1%, *p* = 0.000004). The presence of the 192R allele was related to at least 2-fold higher risk of atherosclerosis requiring PCI/CABG (OR = 2.26, 95% CI: 1.59–3.22; *p* = 0.000004).

The 55MM genotype was more frequent in group A than in group B (29.3% vs. 18.3%; *p* = 0.0304). In carriers of the 55MM genotype, the odds ratio for atherosclerosis requiring PCI/CABG was as high as 1.85 (95% CI: 1.06–3.23; *p* = 0.0304). The 55M allele was more frequent in group A than in group B (45.7% vs. 37.0%; *p* = 0.0350). Its presence in a genotype was shown to slightly increase the risk of atherosclerosis requiring PCI/CABG (OR = 1.44, 95% CI: 1.03–2.01; *p* = 0.0350). After Bonferroni correction, these relationships were not statistically significant.

In this study, the genotype distributions of the Q192R and L55M polymorphisms deviated from Hardy–Weinberg equilibrium in both groups (group A: *p* = 0.000001 and *p* = 0.00006, respectively; group B: *p* = 0.000001 and *p* = 0.0176, respectively).

The frequencies of *PON1* haplotypes comprising the Q192R and L55M polymorphisms in the study and control groups are presented in Table 3. An increased risk of atherosclerosis requiring PCI/CABG was demonstrated in carriers of two haplotypes, i.e., 192R-55L and 192R-55M. Among individuals carrying the 192R-55L haplotype, the odds ratio was 2.06 (95% CI: 1.29–3.28; *p* = 0.0022). In carriers of the 192R-55M haplotype, OR was 1.77 (95% CI: 1.17–2.69; *p* = 0.007). In group A, the 192Q-55L haplotype was less frequent than in group B (33.5% vs. 51.7%). Furthermore, OR for atherosclerosis requiring PCI/CABG in individuals with the 192Q-55L haplotype was 0.47 (95% CI: 0.34–0.66; *p* = 0.000013).

## 3. Discussion

Available data indicate that the *PON1* gene plays a crucial role in lipid metabolism, and thereby in progression of atherosclerosis. Numerous studies have attempted to determine the influence of *PON1* polymorphisms on morbidity due to coronary artery disease (CAD), however, consistent findings have not yet been obtained. Some studies and meta-analyses investigating the *PON1* single nucleotide polymorphisms Q192R and L55M have linked specific *PON1* genotypes to a higher risk of CAD, whereas others have reported weak or non-significant associations, or have found no association at all [10,11,12,13,14,15,16,17,18,19].

A meta-analysis of 64 studies by Hernández-Díaz et al. has shown that carriers of the 192R allele face a lower risk of myocardial infarction (MI) and CAD in Asian and African, though not in European and American populations. Moreover, the presence of the 192Q allele may lead to depletion in HDL and low activity of *PON1*, which consequently does increase the risk of CAD [20].

This study, which was conducted in the Polish population, showed that the presence of the 192RR genotype was associated with a 2.5-fold increase in risk of atherosclerosis requiring PCI/CABG (OR = 2.50, 95% CI: 1.40–4.46; *p* = 0.002). Similarly, the 192R allele was linked to over a two-fold higher risk of atherosclerosis resulting in PCI/CABG (OR = 2.26, 95% CI: 1.59–3.22; *p* = 0.000004) [21].

Furthermore, studies on a relationship between the L55M *PON1* polymorphism and the risk of CAD have inconsistent results, [22,23,24,25,26]. Initially, the L55M polymorphism was considered not to affect *PON1* activity. However, subsequent studies demonstrated that carriers of the 55M allele had both lower levels of *PON1* mRNA and lower plasma *PON1* concentration, which was related to a reduction in serum *PON1* activity by 30% [27]. Across 30 analyzed studies by Hernández-Díaz et al., there was no statistical evidence on any impact of the L55M polymorphism on the cardiovascular risk. Having performed the aforementioned meta-analysis, the authors noted that this risk was higher in European and Asian, but not in African and Mexican populations [20].

The results of the present study indicate that, after Bonferroni correction, there was no statistically significant association between the L55M polymorphism and coronary artery stenosis (Table 2). Nevertheless, before correction for multiple comparisons, carriers of the 55MM genotype exhibited a 1.85-fold higher risk of atherosclerosis requiring PCI/CABG (OR = 1.85, 95% CI: 1.06–3.23; *p* = 0.0304). Similarly, the 55M allele was related to a slightly increased risk of atherosclerosis requiring PCI/CABG (OR = 1.44, 95% CI: 1.03–2.01; *p* = 0.0350).

Because of the discrepancies in scientific reports concerning separate *PON1* single nucleotide polymorphisms, some of research teams have attempted to combine the Q192R and the L55M polymorphisms together as haplotypes. Sanghera et al. observed that the 192R-55L haplotype was related to an increased risk of CAD in Asian Indians (OR = 1.44, 95% CI: 1.08–1.93; *p* = 0.01). The authors suggested that this association may have been largely driven by the effect of the 192R allele since the L55M polymorphism did not appear to influence disease risk [28]. Ahmad et al. demonstrated that the 192R-55M haplotype was the factor responsible for a higher risk of CAD in the Asian population (OR = 5.36, 95% CI: 2.045–14.049; *p* = 0.0001), whereas individuals with the 192Q-55L haplotype were at a lower risk of CAD (OR = 0.73, 95% CI: 0.55–0.97; *p* = 0.03) [29].

Moreno-Godínez et al. analyzed correlations of *PON1* genotypes for the Q192R and L55M polymorphisms with *PON1* activity and CAD in Mexican women. The researchers observed that female carriers of the 55LM and 55MM genotypes were at risk of obesity (OR = 1.6, *p* = 0.039), and the 192Q-55M haplotype was associated with reduced *PON1* activity both in terms of arylesterase (−22.4 U/mL, *p* < 0.001) and paraoxonase (−3.8 U/mL, *p* < 0.001), compared with the 192R-55L haplotype [30].

A study from Italy showed that none of the genotype combinations for the Q192R and L55M polymorphisms were associated with any significant change in the risk of atherosclerosis in the coronary arteries. It was found that carriers of the 192QQ-55MM diplotype showed a lower risk of myocardial infarction than those with the 192RR-55LL diplotype (OR = 0.51, 95% CI: 0.26–0.99; *p* = 0.048) [31].

Gardemann et al. demonstrated that none of the genotype combinations for the Q192R and L55M polymorphisms affected the risk and severity of CAD or risk of MI in the German population [32]. These results are consistent with those obtained by Cascorbi et al. who did not identify any statistically significant differences in the distribution of the haplotypes 192Q-55L, 192R-55L and 192Q-55M in the group of CAD patients compared to the control group (16.9% vs. 17.1%, 6.6% vs. 7.3%, 11.8% vs. 10.3%, respectively). None of the *PON1* haplotypes were related to early disease onset, CAD severity or acute coronary syndromes. Logistic regression analysis adjusted for age, sex, diabetes, arterial hypertension, hypercholesterolemia and smoking did not provide evidence of an increased coronary risk associated with any specific *PON1* genotype [33].

The obtained results indicate that the risk of atherosclerosis requiring PCI/CABG is 2.06-fold for the 192R-55L haplotype and 1.77-fold for the 192R-55M haplotype.

Moreover, another haplotype, i.e., 192Q-55L, was less frequent in patients with atherosclerosis resulting in PCI/CABG than in those without significant plaques in the coronary vessels (OR = 0.47, Table 3). The present study provides evidence that *PON1* haplotypes may play an important role in the development of coronary atherosclerosis. Further research is required to confirm these results and their potential in terms of a clinical setting.

## 4. Materials and Methods

This was a retrospective study including a total of 282 individuals of both sexes. All the participants underwent coronary angiography in 2016–2020, according to guidelines of the European Society of Cardiology (ESC) [34,35,36,37]. The study was also conducted in accordance with the Declaration of Helsinki, and the study protocol was approved by the Bioethics Committee (opinions no. 43/2016 on 12 April 2016 and 63/2019 on 21 May 2019). The inclusion criteria were as follows: eligibility for coronary angiography, age of at least 18 years and written informed consent covering both laboratory and genetic testing. The only exclusion criterion was previous dissection of a coronary vessel. Several factors, including diet, physical activity and medication use, were not taken into account in this study, which represents a limitation. Most of the participants were treated with inhibitors of 3-hydroxy-3-methylglutaryl-coenzyme A reductase.

The Q192R and L55M *PON1* polymorphisms were identified by the restriction fragment length polymorphism–polymerase chain reaction (PCR-RFLP) method based on the procedures developed by Arpaci et al. and Arca et al. [31,38]. Genomic DNA was extracted from peripheral blood leukocytes with GeneMATRIX Quick Blood DNA Purification Kit (EURx Ltd., Molecular Biology Products, Gdańsk, Poland), according to the manufacturer’s protocol. Products obtained in PCR were digested with the *Alw1* restriction enzyme (for the Q192R polymorphism; New England BioLabs, Inc., Ipswich, UK) and with the *Nla III* restriction enzyme (for the L55M polymorphism; New England BioLabs, Inc., Ipswich, UK). The obtained digestion products were separated in 3% agarose gel stained with ethidium bromide.

The frequencies of *PON1* alleles, genotypes and haplotypes in the study group were compared to those in the control group. The frequencies of *PON1* genotypes observed in each group were compared to the expected frequencies, calculated based on the Hardy–Weinberg equation. The χ^2^ test was used to check if the observed differences were statistically significant (*p* < 0.05). The *PON1* haplotypes were reconstructed with PHASE software, version 2.1. In the case of continuous variables (age, components of the lipid profile), the consistency of their distribution with normal distribution and the consistency between group variances were assessed with the Shapiro–Wilk W-test and the Brown–Forsythe test, respectively. Where a specific variable was not normally distributed or lacked homogeneity, the non-parametric Mann–Whitney U-test was used for comparison. When normal distribution and homogeneity of variances were present, the *t*-test for independent samples was used. Empirical *p* values were reported, whereas the direction and magnitude of associations were assessed using odds ratios with 95% confidence intervals. Bonferroni correction was applied to account for multiple comparisons. Calculations were made with TIBCO Statistica, version 13.3 (TIBCO Software Inc.).

## 5. Conclusions

The 192R-55L and 192R-55M haplotypes may be associated with an increased risk of atherosclerosis requiring PCI/CABG, whereas individuals carrying the 192Q-55L haplotype may have a lower susceptibility to CAD. If confirmed in further studies, data on *PON1* haplotypes could be used to identify patients at increased risk of atherosclerosis and to support the implementation of individualized preventive or therapeutic strategies. This, in turn, may contribute to reducing cardiovascular morbidity and mortality.

## Figures and Tables

**Table 1:** Clinical characteristics of the study participants.

	Patients After PCI or Qualified for CABG (Group A), *n* = 140	Patients with no Significant Plaques in Coronary Arteries (Group B), *n* = 142	Statistical Significance of Differences Between the Groups (A vs. B)
Age: mean ± SD (min–max) [yrs.]	65.7 ± 10.0 (45–88)	64.3 ± 10.4 (40–89)	*p* = 0.229
Females, *n* (%) Males, *n* (%)	57 (40.7%) 83 (59.3%)	78 (54.9%) 64 (45.1%)	
Total cholesterol: median (interquartile range: 25th–75th percentile) [mg/dL]	162.5 (138.0–210.0)	171.0 (143.0–203.0)	*p* = 0.609
LDL-C: median (interquartile range: 25th–75th percentile) [mg/dL]	80.0 (61.5–109.0)	84.0 (53.0–106.0)	*p* = 0.492
TG: median (interquartile range: 25th–75th percentile) [mg/dL]	132.0 (86.0–168.0)	110.0 (83.0–172.0)	*p* = 0.144
HDL-C: median (interquartile range: 25th–75th percentile) [mg/dL]	53.0 (46.0–63.0)	58.0 (46.0–74.2)	*p* = 0.023
Non-HDL-C: median (interquartile range: 25th–75th percentile) [mg/dL]	107.0 (86.0–148.0)	112.2 (86.0–136.5)	*p* = 0.595

PCI—percutaneous coronary intervention, CABG—coronary artery bypass graft, SD—standard deviation, LDL-C—low-density lipoprotein cholesterol, TG—triglycerides, HDL-C—high-density lipoprotein cholesterol, non-HDL-C—non-high-density lipoprotein cholesterol, *p*—significance level (after Bonferroni correction, statistically significant differences at *p* < 0.0083).

**Table 2:** The frequency of particular *PON1* genotypes and alleles for the Q192R and L55M polymorphism in the study participants.

***PON1* Genotypes for the Q192R Polymorphism**	**Study Group (A),** ***n* = 140 (%)**	**Control Group (B), *n* = 142 (%)**	**OR**	**95% CI**	***p***
192QQ	56 (40.0)	87 (61.3)	0.42	0.26–0.68	0.0004 *
192QR	40 (28.6)	33 (23.2)	1.32	0.77–2.26	0.307
192RR	44 (31.4)	22 (15.5)	2.50	1.40–4.46	0.002 *
***PON1* alleles for the Q192R polymorphism**	**Study group, *n* = 280 (%)**	**Control group, *n* = 284 (%)**	**OR**	**95% CI**	***p***
192Q	152 (54.3)	207 (72.9)	0.44	0.31–0.63	0.000004 *
192R	128 (45.7)	77 (27.1)	2.26	1.59–3.22	
***PON1* genotypes for the L55M polymorphism**	**Study group, *n* = 140 (%)**	**Control group, *n* = 142 (%)**	**OR**	**95% CI**	***p***
55LL	53 (37.85)	63 (44.4)	0.76	0.47–1.23	0.2667
55LM	46 (32.85)	53 (37.3)	0.82	0.50–1.34	0.4320
55MM	41 (29.3)	26 (18.3)	1.85	1.06–3.23	0.0304
***PON1* alleles for the L55M polymorphism**	**Study group, *n* = 280 (%)**	**Control group, *n* = 284 (%)**	**OR**	**95% CI**	***p***
55L	152 (54.3)	179 (63.0)	0.70	0.50–0.98	0.0350
55M	128 (45.7)	105 (37.0)	1.44	1.03–2.01	

OR—odds ratio, 95% CI—95% confidence interval for OR, *p*—significance level, *—statistically significant differences at *p* < 0.00625 after Bonferroni correction.

**Table 3:** The distribution of particular *PON1* haplotypes for the Q192R and L55M polymorphisms in the study participants.

*PON1* Haplotypes for the Q192R and L55M	Study Group (A), *n* = 280 (freq ± SD)	Control Group (B), *n* = 284 (freq ± SD)	OR	95% CI	*p*
192Q-55L	94 (33.5 ± 0.8)	147 (51.7 ± 1.0)	0.47	0.34–0.66	0.000013 *
192R-55L	58 (20.8 ± 0.8)	32 (11.3 ± 1.0)	2.06	1.29–3.28	0.0022 *
192Q-55M	58 (20.8 ± 0.8)	60 (21.2 ± 1.0)	0.98	0.65–1.46	0.9042
192R-55M	70 (25.0 ± 0.8)	45 (15.8 ± 1.0)	1.77	1.17–2.69	0.007 *

SD—standard deviation for the frequency (freq) of a particular haplotype, OR—odds ratio, 95% CI—95% confidence interval for OR, *p*—significance level, *—statistically significant differences according to the case-control permutation test at *p* < 0.0125 after Bonferroni correction.

## Data Availability

The data presented in this study are available on request from the corresponding author.
